# Associations between overactive bladder and sleep patterns: a cross-sectional study based on 2007–2014 NHANES

**DOI:** 10.1186/s12894-023-01329-z

**Published:** 2023-11-13

**Authors:** Zechao Lu, Jiahao Zhang, Shihao Lin, Zhongxi Fan, Zhaohui He, Fucai Tang

**Affiliations:** 1 Department of Urology, The Eighth Affiliated Hospital, Sun Yat-sen University, 518033, No. 3025, Shennan Zhong Road, Shenzhen, Guangdong 518033 China; 2 The Second Clinical College of Guangzhou Medical University, Guangzhou, Guangdong 511436 China; 3 The Third Clinical College of Guangzhou Medical University, Guangzhou, Guangdong 511436 China

**Keywords:** Overactive bladder, Sleep, Urgent urinary incontinence, Cross-sectional study, NHANES

## Abstract

**Objectives:**

To determine whether relationship exists between overactive bladder (OAB) and sleep patterns through the cross-sectional study.

**Patients and methods:**

Patients from the National Health and Nutrition Examination Survey (NHANES) 2007–2014 were included in this study. Data were extracted through questionnaires, including demographics, dietary and health-related behaviors, body measurements and disease information. Three sleep factors were included to aggregate overall sleep scores, ranging from 0 to 3. A sleep score of 0 to 1, 2 or 3 was expressed as a bad, intermediate or healthy sleep pattern, respectively. The Overactive Bladder Symptom Score (OABSS) scale was applied to quantify the severity of OAB for each participant. Weighted logistic regression models were used to investigate the associations between sleep and OAB.

**Results:**

A total of 16,978 participants were enrolled in this study. The relationship between OAB and sleep patterns was statistically significant. After fully adjusting for confounding factors, the OAB risk of patients with intermediate and poor sleep patterns obviously increased by 26% and 38%, respectively, and mild (OR = 1.21, 95% CI [1.03,1.42]), moderate (OR = 1.45, 95% CI [1.27,1.66]) and severe (OR = 1.57, 95% CI [1.18,2.09]) OAB were significantly associated with sleep pattern grouping. The prevalence of OAB is significantly higher in patients with bad sleep patterns, and vice versa.

**Conclusion:**

This study indicated that there is a positive relationship between OAB and worse sleep-related issues.

**Supplementary Information:**

The online version contains supplementary material available at 10.1186/s12894-023-01329-z.

## Introduction

As part of lower urinary tract symptoms, overactive bladder (OAB) is a group of clinical symptoms characterized by urgent urination, usually accompanied by frequent urination and nocturia, with or without urgent urinary incontinence (UUI), ruling out urinary tract infection or other obvious pathological changes [1]. According to the NOBLE Program in the United States in 2003, the prevalence of OAB in men and women over 18 years old was 16.0% and 16.9%, respectively, the incidence of urge urinary incontinence (UUI) was more common in women, and the incidence of UUI was significantly increased in women after the age of 44 and in men after the age of 64 [2]. Irwin, Debra E et al. noted that the worldwide incidence of OAB in 2008 was estimated at 10.7% [3]. Although no further epidemiological studies have been conducted due to differences in survey methods and definitions, this prevalence was projected to be 20.1% by 2018, since the risk of the disease increased with aging [3–5].

As a functional disease of the bladder, OAB can have a substantial negative effect on quality of life, affecting patients’ physical and mental health [6, 7]. OAB patients’ need to urinate can lead to the disruption of sleep [8]. Other studies have also mentioned the negative impact on quality of sleep among patients diagnosed with OAB [9–11]. Inadequate sleep has a negative influence on general health and well-being [12]. A previous study reported that 49.0% of citizens in Palanga have assessed their sleep quality as fair, rather poor, or poor [13]. Poor sleepers usually experience longer latencies, frequent nocturnal awakenings, less total sleep time, etc., which are common manifestations of OAB [14, 15]. However, the pathogenesis of OAB is not completely clear, and its etiology is multifactorial. A previous study reported that OAB risk factors included age, body mass index, socioeconomic status, diabetes, and smoking [16]. Furthermore, sleep disorders such as obstructive sleep apnea were reported as one of the pathologic factors resulting in OAB [17]. However, it is limited to a finite number of variables.

To address these limitations, we aim to determine whether a relationship exists between overactive bladder (OAB) and sleep patterns through the cross-sectional study based on the large dataset NHANES.

## Patients and methods

### Study population

All data in this study were derived from the National Health and Nutrition Examination Survey (NHANES), conducted by the National Center for Health Statistics of the Centers of Disease Control and Prevention. The data in the NHANES are utilized for epidemiological studies and nutritional health assessments. In this cross-sectional study, the data were collected from NHANES 2007–2014, including the data of participants aged 20–80, as well as complete and reliable information (demographics, dietary and health-related behaviors, body measurements and disease information). A total of 16,978 participants were enlisted in our study. All of these data are publicly available on the NHANES (https://www.cdc.gov/nchs/nhanes/index.htm).

### Assessment of OAB

According to the International Continence Society, OAB is a symptom syndrome characterized by the simultaneity of urinary urgency, usually accompanied by frequency and nocturia, with or without urge urinary incontinence (UUI), without the presence of urinary tract infection or other obvious pathology [6]. To make a diagnosis of exclusion, we considered OAB when patients had relevant clinical manifestations, such as urgency, nocturia and UUI, of which UUI is a specific indicator of urgency [18]. Therefore, one question was used to assess the severity of UUI in our study: “Have you ever urinated before reaching the toilet”, and other question was used to assess the severity of nocturia: “how many times urinate in night?”. Participants were considered to have OAB if they had UUI with/without nocturia (urinate more than twice in one night) when performing some exclusion criteria [18]. The exclusion criteria were as follows: participants with dysuria (n = 186), non-micturition emptiness (n = 137), and benign prostate hypertrophy (n = 165); participants with a history of urinary tract infection by chlamydia, gonococcus and trichomonas (n = 185); participants with cancer history of urinary system, reproductive system and adjacent organs (n = 664), including prostate cancer, bladder cancer kidney cancer, cervical cancer, uterine cancer, colon cancer, and rectum cancer; participants with relevant neurologic disease (n = 663), such as neurologic tumors and stroke; and participants with missing information (n = 1244). The severity of OAB for each participant was quantified through a novel Overactive Bladder Symptom Score (OABSS) scale [19] and classified into none, mild, moderate and high based on questionnaires of UUI and nocturia in participants with OAB.

### Assessment of sleep factors and definition of a sleep pattern

Participant’s response to the question “How much sleep do you usually get at night on weekdays or workdays?” generated the sleep duration, which was classified as short (< 7 h per night), normal (7–9 h per night), and long (> 9 h per night). The assessment of trouble sleeping and sleep disorders were obtained by the response to the question “Have you ever told a doctor or other health professional that you have trouble sleeping?” and “Have you ever been told by a doctor or other health professional that you have a sleep disorder?”, respectively. For each sleep factor (self-reported sleep duration, trouble sleeping and diagnosed sleep disorder), the lower and higher risks were classified as 1 and 0 to aggregate overall sleep scores, ranging from 0 to 3. A sleep score of 0 to 1, 2 or 3 was expressed as a bad, intermediate or healthy sleep pattern, respectively [20].

### Assessment of other covariates

Participants’ self-reported age (20–29, 30–39, 40–49, 50–59, 60–69, 70–80 years), sex (female, male), ethnicity (no-white, white), annual household income (<$20,000, >$20,000), education level (≤ High School, >High School) and marital status (yes/no) at interview were collected from the NHANES demographic files. The recreational activity based on the Global Physical Activity Questionnaire (GPAQ) was classified as yes or no. The sedentary time (< 5 h, ≥ 5 h) was referred to as the duration spent sitting in a typical day, excluding sleeping. Alcohol use, smoking status and self-reported diseases, including diabetes, hypertension and cardiovascular disease, were expressed as yes or no.

### Statistical analysis

The baseline characteristics of the participants were described as proportions for the above categorical variables based on sleep patterns and the severity of OAB. Furthermore, a chi-squared test was performed to compare the baseline characteristics of these participants. Taking into consideration the complicated sampling design of the survey, we applied the survey weight, which permits it to be representative of the U.S. noninstitutionalized civilian [21]. Then, a logistic regression model was adapted to evaluate the association between OAB and sleep. The odds ratio (ORs) and 95% confidence intervals (CI) in OAB patients with different sleep patterns. We also estimated the ORs and 95% CIs of different sleep patterns in patients diagnosed with OAB stratified by age, sex, ethnicity, annual household income, education level, marital status, BMI, sedentary time, smoking status, alcohol use, recreational activity, hypertension, diabetes and cardiovascular disease. A *p* value < 0.05 was considered to be statistically significant. All statistical analyses were carried out using R version 4.2.0.

## Results

### Baseline characteristics of the participants

**Fig. 1:**
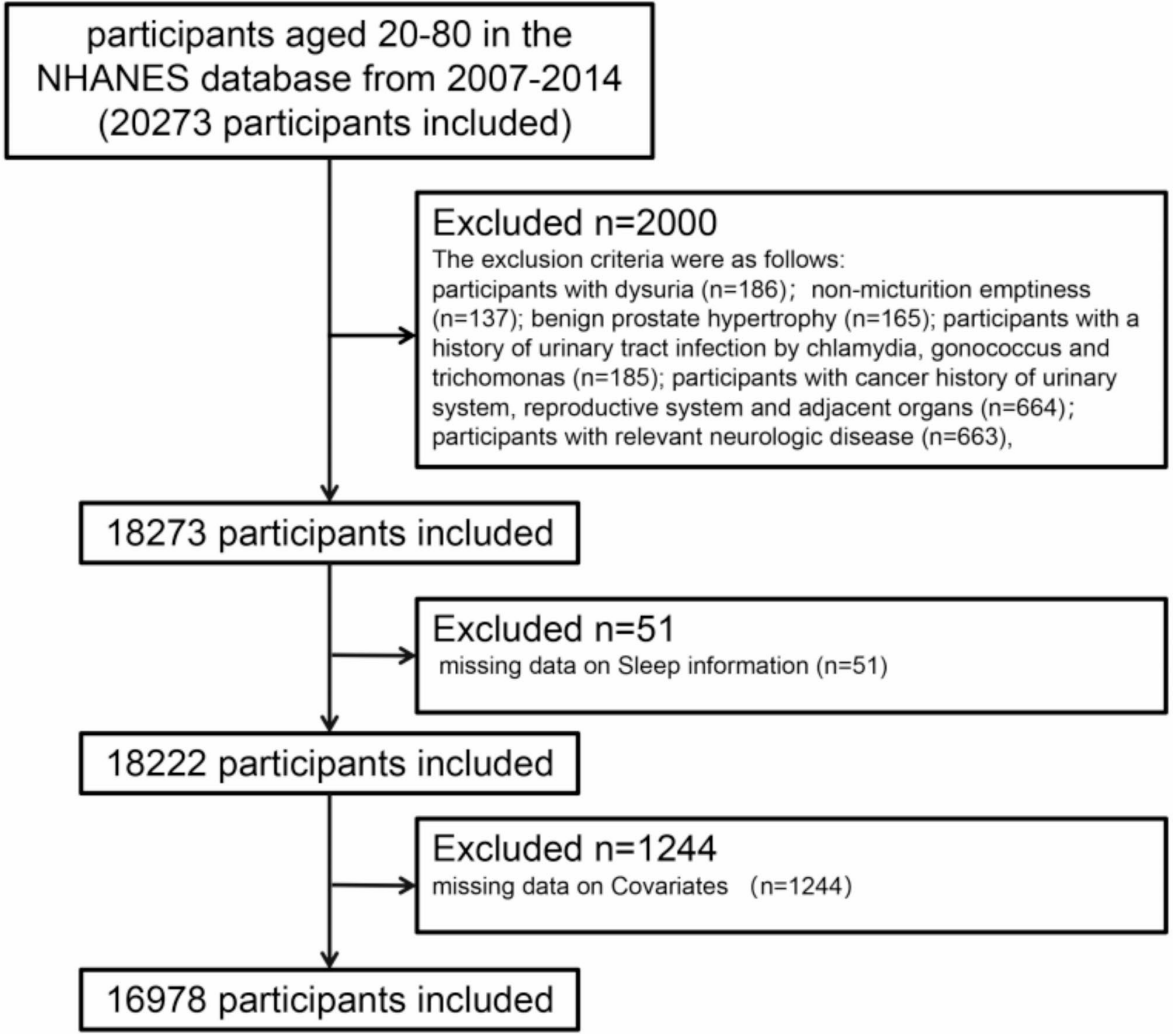
A flowchart depicting the inclusion and exclusion of participants

Figure 1 is A flowchart dissecting the inclusion and exclusion of participants. A total of 16,978 participants were involved in this study representing about 174 million USA adult population (mean [SE] age, 46.4 [0.3] years), of which 3537 participants were considered OAB with UUI representing about 31 million USA population (mean [SE] age, 56.1 [0.4] years). The characteristics of the participants according to OAB (urgency incontinence-based) status are presented in Table 1. There were significant differences between the groups with and without OAB in terms of age, sex, marital status, household income, education, medical comorbidities (such as coronary heart disease, hypertension and diabetes), alcohol use status, BMI, and moderate recreational activities. Compared to participants without OAB, those with OAB had a higher likelihood of being middle-aged or older (50–80), female, married, had a higher BMI, lower income, low education, being inactive in moderate recreational activity, and heavy smoking, and less likely to be alcohol lovers. In particular, sleep factors (sleep pattern grouping, sleep duration grouping, sleep trouble, sleep disorders) were significantly associated with OAB. With the aggravation of OAB, more patients develop unhealthy sleep patterns, sleep trouble and sleep disorders and are prone to abnormal sleep duration. In addition, the prevalence of medical comorbidities such as cardiovascular disease, hypertension and diabetes mellitus was also increasing with higher OABSS (Table 1).

**Table 1:** Baseline characteristics of the study population according to OAB status

Variable	Without OAB	With OAB			P value
		Mild	Moderate	High	
Age (%, SE)					< 0.001
20–29	22.3(0.8)	9.4(1.1)	4.4(0.7)	2.8(1.0)	
30–39	20.2(0.5)	11.4(1.0)	8.0(0.8)	3.4(1.0)	
40–49	20.4(0.5)	20.8(1.3)	12.9(1.2)	10.6(3.0)	
50–59	18.2(0.5)	22.6(1.5)	23.0(1.8)	20.1(3.1)	
60–69	11.4(0.5)	21.4(1.3)	20.9(1.3)	19.7(2.8)	
70–80	7.5(0.3)	14.4(1.0)	30.8(1.7)	43.4(3.4)	
Sex (%, SE)					< 0.001
Female	46.3(0.5)	74.4(1.5)	70.1(1.7)	77.0(2.7)	
Male	53.7(0.5)	25.6(1.5)	29.9(1.7)	23.0(2.7)	
Race (%, SE)					0.026
No-white	30.8(1.7)	28.6(2.1)	33.1(2.4)	36.8(3.7)	
White	69.2(1.7)	71.4(2.1)	66.9(2.4)	63.2(3.7)	
Marital status (%, SE)					< 0.001
No	28.3(0.9)	20.1(1.4)	16.3(1.5)	13.1(2.2)	
Yes	71.7(0.9)	79.9(1.4)	83.7(1.5)	86.9(2.2)	
Annual household income (%, SE)					< 0.001
<20,000	13.2(0.7)	14.3(1.3)	22.7(1.4)	30.0(2.4)	
>20,000	86.8(0.7)	85.7(1.3)	77.3(1.4)	70.0(2.4)	
Education Level (%, SE)					< 0.001
≤High School	37.2(1.2)	37.8(1.8)	49.3(2.4)	56.2(3.3)	
>High School	62.8(1.2)	62.2(1.8)	50.7(2.4)	43.8(3.3)	
Cardiovascular Disease (%, SE)					< 0.001
No	95.4(0.3)	93.0(0.7)	87.0(1.0)	84.1(2.5)	
Yes	4.6(0.3)	7.0(0.7)	13.0(1.0)	15.9(2.5)	
Hypertension (%, SE)					< 0.001
No	67.4(0.7)	59.0(1.7)	38.8(1.7)	30.0(3.0)	
Yes	32.6(0.7)	41.0(1.7)	61.2(1.7)	70.0(3.0)	
Diabetes Mellitus (%, SE)					< 0.001
No	89.3(0.4)	83.6(1.1)	71.3(1.5)	63.0(3.7)	
Yes	10.7(0.4)	16.4(1.1)	28.7(1.5)	37.0(3.7)	
Smoke (%, SE)					0.048
Less	56.5(0.9)	53.8(1.5)	52.9(1.9)	50.4(3.4)	
More	43.5(0.9)	46.2(1.5)	47.1(1.9)	49.6(3.4)	
Alcohol user (%, SE)					< 0.001
No	10.4(0.7)	10.7(1.0)	14.4(1.2)	21.9(2.4)	
Yes	89.6(0.7)	89.3(1.0)	85.6(1.2)	78.1(2.4)	
BMI (%, SE)					< 0.001
<25	32.1(0.7)	26.2(1.7)	19.8(1.2)	18.5(2.8)	
≥25	67.9(0.7)	73.8(1.7)	80.2(1.2)	81.5(2.8)	
Moderate recreational activity (%, SE)					< 0.001
No	42.3(1.0)	52.8(1.9)	59.6(1.9)	75.5(3.4)	
Yes	57.7(1.0)	47.2(1.9)	40.4(1.9)	24.5(3.4)	
Sitting time (%, SE)					0.080
<5	38.1(0.8)	36.5(1.9)	34.6(1.5)	30.7(3.1)	
≥5	61.9(0.8)	63.5(1.9)	65.4(1.5)	69.3(3.1)	
Sleep pattern grouping (%, SE)					< 0.001
Healthy	50.9(0.7)	44.5(1.7)	39.5(1.3)	37.1(3.2)	
Intermediate	33.8(0.6)	36.4(1.7)	35.2(1.4)	33.6(3.6)	
Bad	15.3(0.4)	19.1(1.2)	25.2(1.3)	29.3(3.3)	
Sleep Disorders (%, SE)					< 0.001
No	92.5(0.3)	90.1(1.0)	85.2(1.2)	83.9(2.6)	
Yes	7.5(0.3)	9.9(1.0)	14.8(1.2)	16.1(2.6)	
Sleep Trouble (%, SE)					< 0.001
No	76.0(0.5)	67.1(1.5)	62.5(1.6)	60.0(3.4)	
Yes	24.0(0.5)	32.9(1.5)	37.5(1.6)	40.0(3.4)	
Sleep duration grouping (%, SE)					< 0.001
<7	36.0(0.7)	35.9(1.5)	39.3(1.5)	43.1(3.0)	
7–9	62.2(0.8)	62.1(1.5)	57.8(1.5)	48.8(3.2)	
>9	1.8(0.2)	2.1(0.4)	3.0(0.4)	8.2(1.9)	

The baseline characteristics of the study population with different sleep patterns are listed in Table 2. Compared with participants with healthy sleep patterns, participants with unhealthy sleep patterns appeared to be middle-aged and older (40–69), more frequently female, with higher BMI, lower education and income, heavy smokers and alcohol users, insufficient moderate recreational activities, long sitting time, and higher rates of cardiovascular disease, hypertension and diabetes mellitus. We also found that patients with unhealthy sleep patterns had a higher incidence of OAB, higher OABSS, and nocturia scores than those with healthy sleep patterns.

**Table 2:** Baseline characteristics of the study population according to sleep status

Variable	Healthy	Not Healthy		P value
		Intermediate	Bad	
Age (%, SE)				< 0.001
20–29	22.3(0.9)	19.3(0.9)	11.7(0.8)	
30–39	18.8(0.7)	19.0(0.7)	15.0(0.8)	
40–49	18.0(0.7)	20.6(0.8)	23.6(0.9)	
50–59	16.3(0.5)	20.1(0.9)	24.5(1.1)	
60–69	13.2(0.6)	12.2(0.6)	15.1(0.8)	
70–80	11.5(0.4)	8.8(0.5)	10.1(0.7)	
Sex (%, SE)				< 0.001
Female	50.0(0.7)	50.4(0.8)	55.8(1.1)	
Male	50.0(0.7)	49.6(0.8)	44.2(1.1)	
Race (%, SE)				< 0.001
No-white	30.2(1.7)	33.5(2.0)	26.9(1.8)	
White	69.8(1.7)	66.5(2.0)	73.1(1.8)	
Marital status (%, SE)				0.015
No	27.0(1.0)	27.1(1.2)	23.3(1.2)	
Yes	73.0(1.0)	72.9(1.2)	76.7(1.2)	
Annual household income (%, SE)				< 0.001
<20,000	12.5(0.7)	14.3(0.7)	19.1(1.2)	
>20,000	87.5(0.7)	85.7(0.7)	80.9(1.2)	
Education Level (%, SE)				0.003
≤High School	36.8(1.3)	39.3(1.4)	41.4(1.6)	
>High School	63.2(1.3)	60.7(1.4)	58.6(1.6)	
Cardiovascular Disease (%, SE)				< 0.001
No	95.3(0.3)	95.2(0.4)	89.9(0.7)	
Yes	4.7(0.3)	4.8(0.4)	10.1(0.7)	
Hypertension (%, SE)				< 0.001
No	68.5(1.0)	64.8(0.8)	49.2(1.1)	
Yes	31.5(1.0)	35.2(0.8)	50.8(1.1)	
Diabetes Mellitus (%, SE)				< 0.001
No	89.5(0.5)	87.8(0.6)	78.8(1.1)	
Yes	10.5(0.5)	12.2(0.6)	21.2(1.1)	
Smoke (%, SE)				< 0.001
Less	61.1(0.8)	53.4(1.1)	45.9(1.2)	
More	38.9(0.8)	46.6(1.1)	54.1(1.2)	
Alcohol user (%, SE)				< 0.001
No	12.0(0.6)	10.0(0.8)	9.6(0.8)	
Yes	88.0(0.6)	90.0(0.8)	90.4(0.8)	
BMI (%, SE)				< 0.001
<25	34.0(1.0)	29.3(0.7)	22.4(1.2)	
≥25	66.0(1.0)	70.7(0.7)	77.6(1.2)	
Moderate recreational activity (%, SE)				< 0.001
No	41.3(1.1)	46.1(1.2)	53.9(1.4)	
Yes	58.7(1.1)	53.9(1.2)	46.1(1.4)	
Sitting time (%, SE)				0.002
<5	38.5(1.0)	38.3(0.9)	33.5(1.3)	
≥5	61.5(1.0)	61.7(0.9)	66.5(1.3)	
OAB (%, SE)				< 0.001
No	84.7(0.5)	81.2(0.8)	75.7(0.9)	
Yes	15.3(0.5)	18.8(0.8)	24.3(0.9)	
OABSS (%, SE)				< 0.001
None	84.7(0.5)	81.2(0.8)	75.7(0.9)	
Mild	8.5(0.4)	10.0(0.6)	10.8(0.6)	
Moderate	5.7(0.3)	7.4(0.4)	10.9(0.5)	
High	1.1(0.1)	1.4(0.2)	2.6(0.4)	
Nocturia score (%, SE)				< 0.001
0	38.8(0.9)	38.5(1.0)	27.9(1.1)	
1	41.6(0.9)	38.1(0.9)	38.7(1.2)	
2	13.4(0.4)	14.9(0.5)	18.9(0.9)	
3	6.2(0.3)	8.5(0.5)	14.4(0.7)	

### Association between sleep pattern grouping and OAB

The association between sleep pattern grouping and OAB is shown in Table 3. Our original model (adjusted for no variable) indicated a significant association between sleep pattern grouping and OAB status. This relationship remained significant in the other two models, in which the incidence of OAB increased with worsening sleep patterns. In model 1, compared with healthy patients, patients with unhealthy sleep patterns were more likely to develop OAB, and the probability was increased by 29% and 78% for intermediate and poor sleep patterns, respectively. Intermediate sleep patterns and bad sleep patterns were significantly increased by 31% and 57%, respectively, after adjustment for sex, age, race, marital status, annual household income, and education (model 2). In model 3 (fully adjusted model), after adjusting for all the variables, the intermediate and poor sleep patterns obviously increased by 26% and 38%, respectively. In summary, the incidence of OAB can be higher with poorer sleep quality.

**Table 3:** Associations between sleep pattern grouping and OAB based on the outcome for OAB status

Character	Model 1 OR (95%CI)	Model 2 OR (95%CI)	Model 3 OR (95%CI)
Exposure: sleep pattern grouping			
Healthy	Reference	Reference	Reference
Intermediate	1.29(1.15,1.44)***	1.31(1.16, 1.48)***	1.26(1.11, 1.42)***
Bad	1.78(1.56,2.03)***	1.57(1.35, 1.81)***	1.38(1.20, 1.59)***
P for trend	< 0.001	< 0.001	< 0.001

^a^ Model outcome was OAB (binary: with OAB and without OAB). ***p < 0.001 **p < 0.01 and *p < 0.05

^b^ Model 1: no covariates were adjusted

^c^ Model 2: adjusted for age, sex, ethnicity, marital status, annual household income, and education

^d^ Model 3: adjusted for age, sex, ethnicity, marital status, annual household income, education, cardiovascular disease, hypertension, diabetes mellitus, smoking status, alcohol use, recreational activity, sitting time and BMI.

Table 4 describes the relationship between sleep pattern grouping and the severity of OAB. In model 1, where we did not adjust for any covariates, patients with OAB had sleep problems more frequently than those without OAB, with mild, moderate and high OAB being 1.30, 1.59 and 1.76 times more likely than patients without OAB, respectively. After adjustment for age, sex, race, marital status, annual household income, and education (model 2), patients with all three OAB levels had higher odds of sleep problems than those without OAB, increasing by 26%, 60%, and 82%, respectively. After additional adjustments for cardiovascular disease, hypertension, diabetes, smoking, alcohol consumption, recreational activity, sitting time and BMI (model 3), mild (OR = 1.21, 95% CI [1.03, 1.42]), moderate (OR = 1.45, 95% CI [1.27, 1.66]) and high (OR = 1.57, 95% CI [1.18, 2.09]) OAB were significantly associated with sleep pattern grouping.

Besides, the interaction between sleep pattern group and OAB was not statistically significant (Table S1 and S2). In summary, the association between OAB and sleep pattern grouping is positive significantly.

**Table 4:** Associations between OABSS and sleep pattern grouping based on the outcome for sleep status

Character	Model 1 OR (95%CI)	Model 2 OR (95%CI)	Model 3 OR (95%CI)
Exposure: OABSS			
None	Reference	Reference	Reference
Mild	1.30(1.11,1.51)**	1.26(1.07,1.48)*	1.21(1.03,1.42)*
Moderate	1.59(1.42,1.78)***	1.60(1.40,1.81)***	1.45(1.27,1.66)***
High	1.76(1.34,2.31)***	1.82(1.35,2.43)***	1.57(1.18,2.09)**
P for trend	< 0.001	< 0.001	< 0.001

^a^ Model outcome was sleep status (binary: healthy and not healthy). ***p < 0.001 **p < 0.01 and *p < 0.05

^b^ Model 1: no covariates were adjusted

^c^ Model 2: adjusted for age, sex, ethnicity, marital status, annual household income, and education

^d^ Model 3: adjusted for age, sex, ethnicity, marital status, annual household income, education, cardiovascular disease, hypertension, diabetes mellitus, smoking status, alcohol use, recreational activity, sitting time and BMI.

## Discussion

As a nationwide representative cross-sectional study, it was utilized to assess the association between sleep patterns and OAB. Our results showed that the prevalence of OAB was significantly higher in patients with poor sleep patterns and that the prevalence of worse sleep patterns was significantly higher in OAB patients. That is, OAB patients are more likely to have trouble sleeping than patients without OAB. Likewise, patients with worse sleep patterns are more likely to develop OAB than those without sleep problems. Furthermore, we analysed the association between sleep patterns and OAB by adjusting for age, sex, ethnicity, marital status, household income and education in model 2 and additionally adjusting for cardiovascular disease, hypertension, diabetes, smoking, alcohol use, recreational activities, sedentary time and BMI in model 3. Compared with model 1, the association is still statistically significant. Thus, it is reasonable to suggest that OAB promotes the occurrence of worse sleep patterns and that worse sleep patterns promote OAB occurrence.

Our results were consistent with those of previous studies. A previous cross-sectional study reported that OAB patients have an elevated risk of insomnia compared with non-OAB individuals [22]. According to a randomized controlled trial, the higher the frequency of urinary incontinence (UUI), the greater the degree of sleep dysfunction [23], which is in line with our finding showing that compared with patients without OAB, those with mild, moderate, and severe OAB were 1.30 times, 1.59 times, and 1.76 times more likely to have sleep problems, respectively. UUI is the representative symptom of OAB [18]. We speculate that the increasing severity of the UUI appears to be associated with the shorter sleep duration of patients and more trouble falling asleep, which leads to the gradual deterioration of sleep quality. Another finding in our study was that a higher incidence of OAB was associated with poorer sleep quality. This was also supported by a prospective questionnaire study. The incidence of OAB symptoms in patients with moderate and severe OSAS (obstructive sleep apnea syndrome) was significantly higher than that in patients with mild OSAS and UARS (upper airway resistance syndrome), and the incidence of UUI in patients with severe OSAS was higher than that in patients with mild OSAS and UARS [17]. A previous review has also suggested that a variety of pathologic factors contribute to nocturia, including sleep-related disorders such as sleep apnea and primary sleep disorders [24]. We hypothesized that sleep-related disorders might be involved in the pathological mechanisms of occurrence and development of OAB.

The pathophysiology between OAB and poor sleep patterns is still unclear. We speculate that there may be potential mechanisms that underlie the relationship between OAB and sleep patterns. First, as one of the common symptoms of OAB, nocturia itself can have an adverse impact on the quality of sleep since OAB patients get up at night more often and spend less time sleeping [25]. Second, obstructive sleep apnea syndrome (OSAS) may be a potential mechanism for sleep patterns affecting OAB. OSAS is a syndrome of repeated apnea accompanied by intermittent oxygen saturation reduction due to upper airway obstruction, which inevitably leads to poor sleep quality [26]. At the same time, OSAS can lead to an increase in the secretion of atrial natriuretic peptide (ANP), which can increase glucose filtration through the renin angiotensin aldosterone system and finally result in nocturnal polyuria [27]. animal experiments have shown that oxidative stress caused by intermittent hypoxia in obstructive sleep apnea is associated with structural and functional changes in the bladder, ultimately giving rise to unstable bladder contractions and increased nocturia frequency [28]. It has been suggested that hypoxia can lead to axonal damage in peripheral nerves, and when this effect is exerted on the nerves that innervate the bladder, OAB may be caused [29]. This indicates that OSAS is related to OAB. Based on the above studies, we speculate that OSAS may lead to poor sleep patterns and lead to OAB by increasing ANP secretion, hypoxia, oxidative stress, and other processes. Finally, there are differences between OAB patients and non OAB patients in multiple organ systems, such as the nervous system, cardiopulmonary system, gastrointestinal system, etc. Many OAB patients have extensive systemic symptoms and multiple organ burden. The burden of these multiple organs leads to poor sleep patterns [30].

There are some strengths in this present study. First, to the best of our knowledge, this study is the first cross-sectional study to investigate the relationship between OAB and sleep patterns based on the NHANES database. Simultaneously, our study is nationally representative because it includes a large sample size of patients living in the United States. Finally, we further guarantee the accuracy of our results by performing adjusted models. In this study, both the unadjusted model and the adjusted models show consistent results, even though some differences were observed.

However, it should be noted that there are several limitations in this current study. First, this study was a cross-sectional study and could not identify the causality and pathological mechanisms associated with OAB and poor sleep patterns, making longitudinal follow-up studies impossible but only confirming a relationship between them. Second, the data in this study were obtained from the self-report questionnaires of patients in the NHANES database, which may lead to recall bias. For example, patients may provide incorrect information due to their vague memory when responding to the questionnaire about the duration of sleep per night. In addition, we redefined the diagnosis of OAB in this study, but it must be admitted that information about OAB in the NHANES dataset is not complete. Finally, insufficient data about the sequence between the diagnosis of OAB and the occurrence of bad sleep patterns limited our further accurate analysis.

In summary, the study indicated that there is a positive relationship between OAB and worse sleep-related issues. Our results showed that the prevalence of OAB was significantly higher in patients with poor sleep patterns and that the prevalence of worse sleep patterns was significantly higher in OAB patients. These findings suggest that further prospective studies should be conducted to investigate the causality and pathological mechanisms between sleep and OAB.

### Electronic supplementary material

Below is the link to the electronic supplementary material.

Supplementary Material 1

Supplementary Material 2

## Data Availability

All of the data are publicly available on the NHANES (https://www.cdc.gov/nchs/nhanes/index.htm).

## References

[1] White N, Iglesia CB, Overactive Bladder (2016). OBSTET GYN CLIN N AM.

[2] Stewart WF, Van Rooyen JB, Cundiff GW (2003). Prevalence and burden of overactive bladder in the United States. WORLD J UROL.

[3] Irwin DE, Kopp ZS, Agatep B, Milsom I, Abrams P (2011). Worldwide prevalence estimates of lower urinary tract symptoms, overactive bladder, urinary incontinence and bladder outlet obstruction. BJU INT.

[4] Coyne KS, Sexton CC, Vats V, Thompson C, Kopp ZS, Milsom I (2011). National community prevalence of overactive bladder in the United States stratified by sex and age. UROLOGY.

[5] Milsom I, Abrams P, Cardozo L, Roberts RG, Thüroff J, Wein AJ (2001). How widespread are the symptoms of an overactive bladder and how are they managed? A population-based prevalence study. BJU INT.

[6] Henderson E, Drake M (2010). Overactive bladder. MATURITAS.

[7] Wang Y, Xu K, Hu H (2011). Prevalence, risk factors, and impact on health related quality of life of overactive bladder in China. NEUROUROL URODYNAM.

[8] Golabek T, Skalski M, Przydacz M (2016). Lower urinary tract symptoms, nocturia and overactive bladder in patients with depression and anxiety. PSYCHIATR POL.

[9] Epstein LB, Goldberg RP (2005). The overactive bladder and quality of life. Int J Fertil Women’s Med.

[10] Kinsey D, Pretorius S, Glover L, Alexander T (2016). The psychological impact of overactive bladder: a systematic review. J HEALTH PSYCHOL.

[11] Tubaro A (2004). Defining overactive bladder: epidemiology and burden of disease. UROLOGY.

[12] Alvarez GG, Ayas NT (2004). The impact of daily sleep duration on health: a review of the literature. Prog Cardiovasc Nurs.

[13] Andruskiene J, Varoneckas G, Martinkenas A, Grabauskas V (2008). Factors associated with poor sleep and health-related quality of life. Med (Kaunas).

[14] Middelkoop HA, Smilde-van den Doel DA, Neven AK, Kamphuisen HA, Springer CP (1996). Subjective sleep characteristics of 1,485 males and females aged 50–93: effects of sex and age, and factors related to self-evaluated quality of sleep. J Gerontol A.

[15] Brown JS, McGhan WF, Chokroverty S (2000). Comorbidities associated with overactive bladder. Am J Manag Care.

[16] Mckellar K, Bellin E, Schoenbaum E, Abraham N, Prevalence (2019). Risk factors, and treatment for overactive bladder in a racially diverse Population. Urology.

[17] Kemmer H, Mathes AM, Dilk O, Gröschel A, Grass C, Stöckle M (2009). Obstructive sleep apnea syndrome is associated with overactive bladder and urgency incontinence in men. Sleep.

[18] Yang L, Liu Z, Peng Z (2022). Exposure to DEHP is potential to increase the risk of overactive bladder, evidence from NHANES 2003–2008. Environ Sci Pollut Res Int.

[19] Blaivas JG, Panagopoulos G, Weiss JP, Somaroo C (2007). Validation of the overactive bladder symptom score. J Urol.

[20] Chunnan L, Shaomei S, Wannian L (2022). The association between sleep and depressive symptoms in US adults: data from the NHANES (2007–2014). EPIDEMIOL PSYCH SCI.

[21] Chen T, Clark J, Riddles MK, Mohadjer LK, Fakhouri THI (2020). National Health and Nutrition Examination Survey, 2015–2018: Sample Design and Estimation Procedures. Vital Health Stat 2.

[22] Przydacz M, Golabek T, Dudek P (2021). Overactive bladder symptoms negatively affect sleep quality of patients with Depression. INT NEUROUROL J.

[23] Winkelman WD, Warsi A, Huang AJ (2018). Sleep quality and daytime sleepiness among women with urgency predominant urinary incontinence. Female Pelvic Medicine & Reconstructive Surgery.

[24] Weiss JP, Blaivas JG (2002). Nocturnal polyuria versus overactive bladder in nocturia. Urology.

[25] Leslie SW, Sajjad H, Singh S (2022). Nocturia. *StatPearls*.

[26] Lévy P, Kohler M, McNicholas WT (2015). Obstructive sleep apnoea syndrome. Nat Reviews Disease Primers.

[27] Umlauf MG, Chasens ER, Greevy RA, Arnold J, Burgio KL, Pillion DJ (2004). Obstructive sleep apnea, nocturia and polyuria in older adults. Sleep.

[28] Witthaus MW, Nipa F, Yang J, Li Y, Lerner LB, Azadzoi KM (2015). Bladder oxidative stress in sleep apnea contributes to detrusor instability and nocturia. J Urol.

[29] Lüdemann P, Dziewas R, Sörös P, Happe S, Frese A (2001). Axonal polyneuropathy in obstructive sleep apnoea. J Neurol Neurosurg Psychiatry.

[30] Lai HH, Vetter J, Jain S, Andriole GL (2016). Systemic nonurological symptoms in patients with overactive bladder. J Urol.

