# Supplementary material for: Associations between overactive bladder and sleep patterns: a cross-sectional study based on 2007–2014 NHANES
Source: BMC Urol. 2023 Nov 13;23:184. doi: 10.1186/s12894-023-01329-z (PMC10642019; doi:10.1186/s12894-023-01329-z)
Supplement: Supplementary file 1 — Supplementary Material 1 [file 12894_2023_1329_MOESM1_ESM.docx]

**Table S2. The subgroup analysis and interactive effect based on OABSS.**

| Character | None | Mild | Moderate | High | P for trend | P for interaction |
| --- | --- | --- | --- | --- | --- | --- |
| Age (%, SE) |  |  |  |  |  | 0.58 |
| 20-29 | Reference | 1.02(0.63,1.64) | 1.72(0.92,3.22) | 0.97(0.23,4.17) | 0.2 |  |
| 30-39 | Reference | 1.28(0.92,1.77) | 1.58(1.00,2.50) | 0.53(0.18,1.52) | 0.04 |  |
| 40-49 | Reference | 1.32(1.00,1.75) | 1.63(1.00,2.67) | 1.66(0.78,3.51) | 0.004 |  |
| 50-59 | Reference | 1.12(0.74,1.69) | 1.40(1.00,1.95) | 3.04(1.28,7.25)* | 0.01 |  |
| 60-69 | Reference | 1.26(0.95,1.68) | 1.69(1.22,2.34)** | 1.23(0.64,2.34) | 0.001 |  |
| 70-80 | Reference | 1.15(0.91,1.46) | 1.15(0.91,1.46) | 1.51(1.06,2.15)* | 0.03 |  |
| Sex (%, SE) |  |  |  |  |  | 0.12 |
| Female | Reference | 1.29(1.04,1.60)* | 1.43(1.21,1.68)*** | 1.43(1.04,1.98)* | <0.0001 |  |
| Male | Reference | 0.99(0.79,1.26) | 1.47(1.14,1.91)** | 1.79(0.89,3.62) | 0.01 |  |
| Race (%, SE) |  |  |  |  |  | 0.87 |
| No-white | Reference | 1.21(1.00,1.47) | 1.42(1.18,1.72)*** | 1.40(1.03,1.90)* | <0.001 |  |
| White | Reference | 1.22(1.00,1.50) | 1.48(1.21,1.81)*** | 1.68(1.16,2.43)* | <0.0001 |  |
| Marital status (%, SE) |  |  |  |  |  | 0.65 |
| No | Reference | 1.09(0.79,1.50) | 1.45(1.06,1.99)* | 1.20(0.66,2.17) | 0.05 |  |
| Yes | Reference | 1.24(1.05,1.46)* | 1.44(1.24,1.69)*** | 1.62(1.19,2.19)** | <0.0001 |  |
| Annual household income (%, SE) |  |  |  |  |  | 0.13 |
| <20,000 | Reference | 0.82(0.64,1.05) | 1.46(1.12,1.91)* | 1.37(0.93,2.01) | 0.01 |  |
| >20,000 | Reference | 1.28(1.08,1.53)* | 1.43(1.21,1.69)*** | 1.62(1.12,2.36)* | <0.0001 |  |
| Education Level  (%, SE) |  |  |  |  |  | 0.88 |
| ≤High School | Reference | 1.12(0.92,1.38) | 1.46(1.21,1.76)*** | 1.53(1.07,2.20)* | <0.0001 |  |
| >High School | Reference | 1.27(1.04,1.57)* | 1.46(1.16,1.82)*** | 1.65(1.04,2.62)* | <0.001 |  |
| Cardiovascular Disease (%, SE) |  |  |  |  |  | 0.29 |
| No | Reference | 1.19(1.01,1.40)* | 1.45(1.26,1.68)*** | 1.45(1.08,1.95)* | <0.0001 |  |
| Yes | Reference | 1.48(0.85, 2.56) | 1.42(0.91, 2.19) | 2.52(1.42, 4.46)** | 0.01 |  |
| Hypertension (%, SE) |  |  |  |  |  | 0.79 |
| No | Reference | 1.14(0.94,1.38) | 1.38(1.10,1.73)* | 1.43(0.84,2.44) | 0.002 |  |
| Yes | Reference | 1.33(1.08,1.63)* | 1.51(1.23,1.85)*** | 1.65(1.16,2.33)* | <0.0001 |  |
| Diabetes Mellitus (%, SE) |  |  |  |  |  | 0.74 |
| No | Reference | 1.20(1.00,1.44) | 1.40(1.19,1.64)*** | 1.64(1.16,2.32)* | <0.0001 |  |
| Yes | Reference | 1.27(0.92,1.75) | 1.62(1.27,2.06)*** | 1.47(0.85,2.54) | 0.002 |  |
| Smoke (%, SE) |  |  |  |  |  | 0.13 |
| Less | Reference | 1.34(1.09,1.65)* | 1.49(1.24,1.79)*** | 1.64(1.14,2.35)* | <0.0001 |  |
| More | Reference | 1.07(0.84,1.35) | 1.37(1.10,1.71)* | 1.55(1.02,2.37)* | 0.002 |  |
| Alcohol user (%, SE) |  |  |  |  |  | 0.61 |
| No | Reference | 1.21(1.02,1.43)* | 1.48(1.27,1.73)*** | 1.67(1.16,2.39)* | <0.0001 |  |
| Yes | Reference | 1.17(0.82,1.65) | 1.25(0.90,1.73) | 1.32(0.74,2.38) | 0.1 |  |
| BMI (%, SE) |  |  |  |  |  | 0.07 |
| <25 | Reference | 1.02(0.73,1.41) | 0.96(0.66,1.40) | 1.95(1.05,3.63)* | 0.52 |  |
| ≥25 | Reference | 1.28(1.07,1.54)* | 1.62(1.39,1.89)*** | 1.52(1.09,2.13)* | <0.0001 |  |
| Moderate recreational activity (%, SE) |  |  |  |  |  | 0.71 |
| No | Reference | 1.12(0.92,1.36) | 1.50(1.24,1.80)*** | 1.49(1.08,2.07)* | <0.0001 |  |
| Yes | Reference | 1.30(1.06,1.58)* | 1.39(1.05,1.84)* | 1.84(1.02,3.33)* | <0.001 |  |
| Sitting time (%, SE) |  |  |  |  |  | 0.10 |
| <5 | Reference | 1.13(0.88,1.47) | 1.24(0.99,1.55) | 0.97(0.65,1.44) | 0.05 |  |
| ≥5 | Reference | 1.26(1.05,1.52)* | 1.59(1.31,1.92)*** | 2.00(1.39,2.88)*** | <0.0001 |  |

^1^ ***p < 0.001 **p < 0.01 and *p <0.05.
