# Supplementary material for: Associations between overactive bladder and sleep patterns: a cross-sectional study based on 2007–2014 NHANES
Source: BMC Urol. 2023 Nov 13;23:184. doi: 10.1186/s12894-023-01329-z (PMC10642019; doi:10.1186/s12894-023-01329-z)
Supplement: Supplementary file 2 — Supplementary Material 2 [file 12894_2023_1329_MOESM2_ESM.docx]

**Table S1. The subgroup analysis and interactive effect based on sleep pattern grouping.**

| Character | Healthy | Intermediate | Bad | P for trend | P for interaction |
| --- | --- | --- | --- | --- | --- |
| Age (%, SE) |  |  |  |  | 0.77 |
| 20-29 | Reference | 1.01(0.68, 1.51) | 1.58(0.94, 2.68) | 0.17 |  |
| 30-39 | Reference | 1.27(0.93,1.71) | 1.45(0.91,2.30) | 0.06 |  |
| 40-49 | Reference | 1.45(1.10,1.89)* | 1.31(0.97,1.78) | 0.02 |  |
| 50-59 | Reference | 1.24(0.89,1.72) | 1.38(0.96,1.96) | 0.07 |  |
| 60-69 | Reference | 1.38(1.07,1.77)* | 1.49(1.11,2.01)* | 0.002 |  |
| 70-80 | Reference | 1.15(0.95,1.41) | 1.29(1.01,1.67)* | 0.03 |  |
| Sex (%, SE) |  |  |  |  | 0.19 |
| Female | Reference | 1.31(1.13,1.53)*** | 1.45(1.20,1.75)*** | <0.001 |  |
| Male | Reference | 1.17(0.98, 1.39) | 1.27(0.96, 1.67) | 0.05 |  |
| Race (%, SE) |  |  |  |  | 0.32 |
| No-white | Reference | 1.20(1.03,1.39)* | 1.52(1.27,1.83)*** | <0.001 |  |
| White | Reference | 1.30(1.10, 1.54)** | 1.35(1.11, 1.64)** | <0.001 |  |
| Marital status (%, SE) |  |  |  |  | 0.63 |
| No | Reference | 1.16(0.89, 1.51) | 1.25(0.91, 1.72) | 0.14 |  |
| Yes | Reference | 1.28(1.12, 1.47)*** | 1.40(1.21, 1.63)*** | <0.001 |  |
| Annual household income (%, SE) |  |  |  |  | 0.07 |
| <20,000 | Reference | 0.98(0.79, 1.21) | 1.34(1.09, 1.64)* | 0.01 |  |
| >20,000 | Reference | 1.32(1.15, 1.52)*** | 1.38(1.16, 1.63)*** | <0.001 |  |
| Education Level  (%, SE) |  |  |  |  | 0.66 |
| ≤High School | Reference | 1.26(1.07, 1.47)* | 1.32(1.16, 1.51)*** | <0.001 |  |
| >High School | Reference | 1.26(1.05, 1.52)* | 1.44(1.16, 1.79)** | <0.001 |  |
| Cardiovascular Disease (%, SE) |  |  |  |  | 0.61 |
| No | Reference | 1.24(1.10, 1.40)*** | 1.36(1.18, 1.57)*** | <0.001 |  |
| Yes | Reference | 1.49(0.95, 2.33) | 1.60(1.02, 2.49)* | 0.03 |  |
| Hypertension (%, SE) |  |  |  |  | 0.15 |
| No | Reference | 1.22(1.04, 1.43)* | 1.16(0.96, 1.42) | 0.03 |  |
| Yes | Reference | 1.32(1.10, 1.58)** | 1.57(1.30, 1.91)*** | <0.001 |  |
| Diabetes Mellitus (%, SE) |  |  |  |  | 0.52 |
| No | Reference | 1.23(1.08, 1.41)** | 1.33(1.14, 1.56)*** | <0.001 |  |
| Yes | Reference | 1.39(1.05, 1.82)* | 1.56(1.17, 2.07)** | 0.002 |  |
| Smoke (%, SE) |  |  |  |  | 0.33 |
| Less | Reference | 1.35(1.15, 1.59)*** | 1.47(1.20, 1.80)*** | <0.001 |  |
| More | Reference | 1.16(0.96, 1.39) | 1.28(1.03, 1.58)* | 0.02 |  |
| Alcohol user (%, SE) |  |  |  |  | 0.85 |
| No | Reference | 1.27(1.13, 1.43)*** | 1.38(1.17, 1.63)*** | <0.001 |  |
| Yes | Reference | 1.15(0.84, 1.59) | 1.31(0.98, 1.75)* | 0.06 |  |
| BMI (%, SE) |  |  |  |  | 0.05 |
| <25 | Reference | 1.04(0.79, 1.39) | 0.94(0.65, 1.35) | 0.89 |  |
| ≥25 | Reference | 1.34(1.16, 1.55)*** | 1.53(1.32, 1.78)*** | <0.001 |  |
| Moderate recreational activity (%, SE) |  |  |  |  | 0.51 |
| No | Reference | 1.27(1.11, 1.47)** | 1.29(1.07, 1.56)* | 0.003 |  |
| Yes | Reference | 1.24(1.04, 1.48)* | 1.50(1.21, 1.86)*** | <0.001 |  |
| Sitting time (%, SE) |  |  |  |  | 0.08 |
| <5 | Reference | 1.13(0.93, 1.37) | 1.12(0.87, 1.44) | 0.23 |  |
| ≥5 | Reference | 1.35(1.17, 1.56)*** | 1.53(1.27, 1.85)*** | <0.001 |  |

^1^ ***p < 0.001 **p < 0.01 and *p <0.05.
